# Intravesical Condyloma Acuminata Progressing to Squamous Cell Carcinoma of the Bladder: An Unusual Presentation

**DOI:** 10.7759/cureus.18259

**Published:** 2021-09-24

**Authors:** Franchesca F. Espinal Alvarez, Walter Marquez Lavenant, Laura I Mendez Morente, Alexandra Ahumada Palma

**Affiliations:** 1 Internal Medicine, Instituto Tecnologico de Santo Domingo, Santo Domingo, DOM; 2 Public Health, University of Nebraska Medical Center, Omaha, USA; 3 Internal Medicine, Mount Sinai Medical Center, Miami, USA; 4 Internal Medicine, Ascension Via Christi St. Francis Hospital, Wichita, USA

**Keywords:** human papillomavirus (hpv), condyloma acuminata, squamous cell carcinoma, scc, bladder tumor, pelvic mass

## Abstract

Squamous cell carcinoma (SCC) of the bladder resulting from condyloma acuminata (CA) is uncommon. Most cases of SCC are asymptomatic until an advanced stage making the diagnosis difficult. Most patients present with urinary symptoms. We present the case of a 31-year-old African American male who presented to the emergency department with right lower extremity deep vein thrombosis. His past medical history was significant for recurrent bladder and urethral CA with high-grade dysplasia. A computed tomography (CT) gave the incidental findings of pelvic and bladder masses. The masses were studied and came back concerning for malignancy. The patient will undergo surgical removal soon. This manuscript illustrates an unusual presentation of CA progressing to SCC, including the diagnostic approach and treatment. We hope to increase awareness of the clinical presentation and regular follow-up in patients with risk factors.

## Introduction

Infection with human papillomavirus (HPV) is considered the most common viral sexually transmitted disease globally, which, according to the Centers for Disease Control and Prevention (CDC), includes 79 million Americans [1]. In the United States, squamous cell carcinoma (SCC) of the bladder comprises less than 5% of all bladder cancers [2]. Condyloma acuminata (CA) presents as a warty lesion resulting from infection by the HPV, especially high-risk types (16, 18, 45 and 56) [3]. It is a benign disease, and its highest prevalence occurs in women age 25 to 35 years old and men age 35 to 44 years old [4]. In men, the lesions present in the external genitalia, although very rare, can extend into the urethra or urinary bladder and develop into cancer. It is rare for CA to occur in the urinary bladder, but its presence is considered a risk factor for SCC [5]. We present the case of a young male with a history of CA with high-grade dysplasia that progressed from CA to urethral and bladder SCC. 

## Case presentation

**Figure 1:**
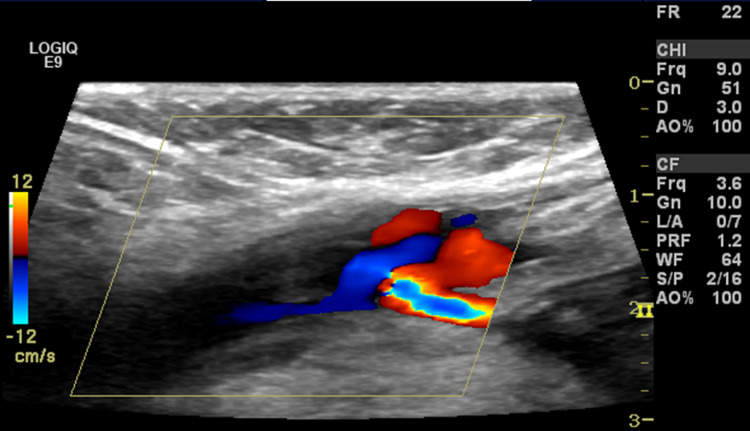
Doppler of the right great saphenous vein. Occlusive deep venous thrombosis in the right common femoral vein extending into the greater saphenous vein.

A 31-year-old African American male presents to the emergency department (ED) with right lower extremity edema over the last three days. His past medical history was significant for recurrent bladder and urethral CA with high-grade dysplasia since age 14 due to HPV, requiring three cystoscopies during his lifetime and 15 years of smoking history. During childhood, he acquired HPV from sexual transmission. Evaluation in the ED revealed right lower limb edema during the review of systems, denying any urinary symptoms. The physical exam was complimentary for right lower extremity edema extending to the mid-thigh with normal radial and posterior tibial pulses. The rest of the exam, including vital signs, was unremarkable. Laboratory workup showed mild leukocytosis of 11.9 k/µL; COVID-19 was negative. An ultrasound of the right lower extremity illustrated deep venous thrombosis (DVT). A nearly occlusive clot within the common femoral vein extended into the greater saphenous vein (Figure 1). A heparin drip was started during the ED course. The patient was admitted under internal medicine with a plan to consult interventional radiology (IR) for catheter-directed tPA on the next day.

**Figure 2:**
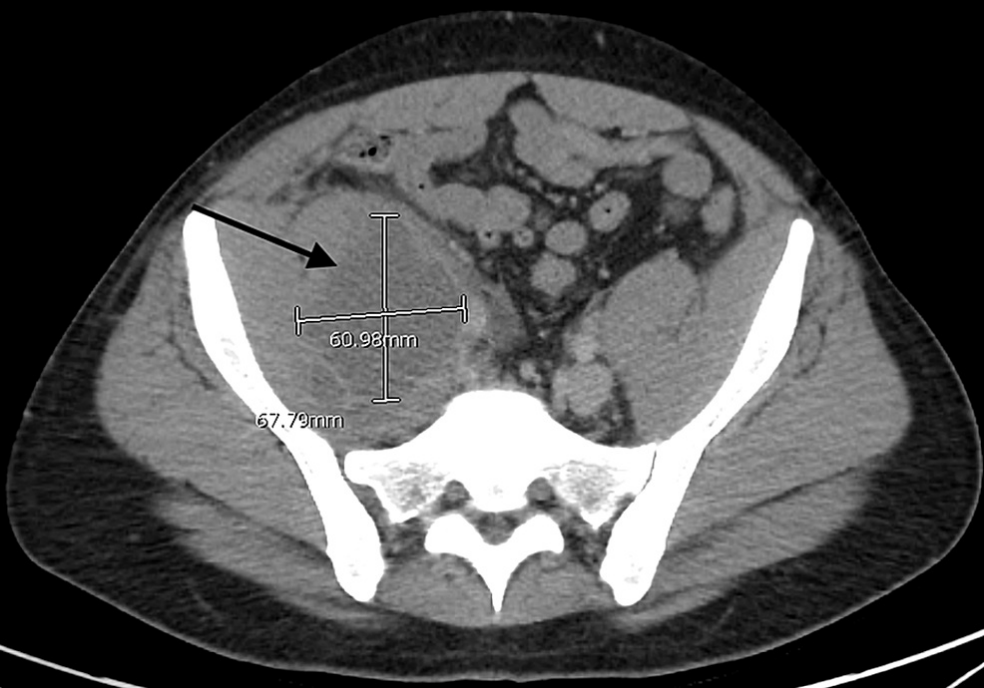
Right-sided pelvic mass within the iliopsoas muscle extending to the pelvic sidewall.

**Figure 3:**
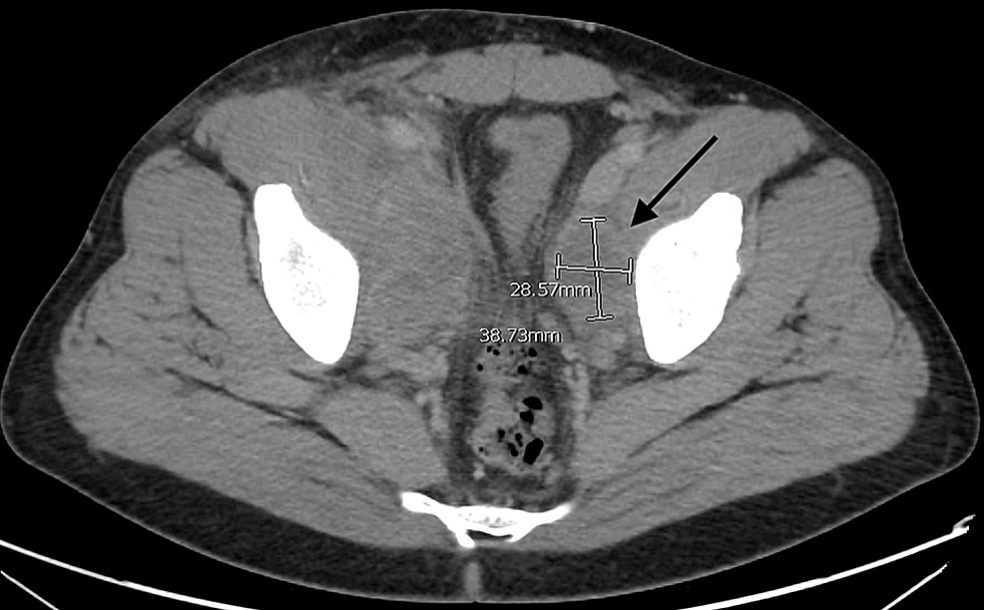
Left-sided pelvic mass within the iliopsoas muscle of 2.8 cm. A nodule with a small cyst.

The following day, he underwent catheter-guided thrombolysis in the right popliteal vein. The catheter remained in place for 24 hours. The IR team ordered a computed tomography (CT)-guided catheter removal. The abdomen CT revealed bilateral pelvic masses within the iliopsoas muscle more pronounced on the right side than the left (Figures 2, 3). Differential considerations included abscesses, lymphocele, and less likely a hematoma. A neoplasm was not excluded. Mild right-sided hydronephrosis and hydroureter secondary to extrinsic compression from the right iliopsoas pelvic mass were noted (Figure 2). Additionally, a 2-cm cystic and solid bladder mass in the urinary bladder's dependent portion was noted (Figure 3). The mass was associated with an extraluminal nodule, measuring 1.5 cm.

The suspicion of abscess with the initial leukocytosis triggered an infectious disease consult and the subsequent initiation of piperacillin-tazobactam. HIV was negative. After an interdisciplinary meeting with urology, infectious disease, and oncology, we decided to perform an IR-guided biopsy and drainage of masses in the right pelvis to identify the etiology; 150 mL of purulent fluid was removed and sent to the laboratory for analysis, and a catheter was left in place for additional decompression, and the heparin drip stayed on given these findings. The patient was notified of the imaging results. Cytology reported numerous neutrophils, necrotic tissue consistent with abscess, and atypical squamous cells that suggested a metaplastic or dysplastic process.

Urology recommended transurethral resection of the urethral and bladder tumors. The procedure was uneventful. After a couple of days, the pathology report returned positive SCC suspected of invading the muscularis propria. The malignancy results were discussed with the patient and his family members. The urology department explained the future surgical plans after discharge for removal of his bladder and prostate and the need to establish care with uro-oncology. The patient experienced postoperative hematuria that resolved after a couple of days. Once stabilized, he was discharged with apixaban and intravenous antibiotics. Urology and infectious disease follow-ups were ordered. The right pelvic mass drain was left in at the time of discharge. On outpatient follow-up, the patient has established care with oncology, who recommended against adjuvant chemotherapy, and urology decided to continue with the surgical plan.

## Discussion

The lesion of condyloma acuminatum results from the infection with HPV [6]. It is uncommon that the lesions extend into the urinary tract and even rarer to spread into the urinary bladder. When SCC develops within the urinary tract, the clinical manifestations are exceptionally varied [2]. Lagwinski et al. examined a series of 45 patients who underwent radical and partial cystectomies for SCC [2]. They described clinical presentations that included hematuria dysuria, flank or suprapubic pain, urgency, frequency, urinary obstruction, and recurrent urinary tract infection. On cystoscopy, cancer can present as an extensive erythematous plaque or as a localized, pale, and hard to visualize lesion [7].

Most cases of SCC are asymptomatic until an advanced stage, however, patients with irritative symptoms could be diagnosed earlier [8]. Microscopic hematuria is the most commonly associated symptom of urinary bladder SCC [9]. Less frequently, the patient might present with dysuria, frequency, nocturia, pelvic pain, or obstructive symptoms, all of which suggest advanced disease. A cystoscopy is a typical approach for asymptomatic gross or microscopic hematuria and physical findings suggesting a more benign etiology (i.e., urolithiasis). Cystoscopy is also the gold standard diagnostic test for a patient with a suspicion of bladder neoplasm, along with deep tissue biopsy. A solitary tumor, extensive and associated leukoplakia, is frequently encountered during the procedure, with the tumor having a preference for the trigone and lateral walls. Although the incidence of distant metastasis is low in the bladder's SCC, the prognosis is poor if present. Most patients die after not getting adequate locoregional control [10]. SCC of the bladder is staged using the American Joint Committee on Cancer (AJCC)/tumor, node, metastasis (TNM) system. This tumor spreads by direct extension to the adjacent organs and by lymph vascular invasion [11]. Imaging such as CT or MRI acts as an adjunct to cystoscopy with deep biopsy to accurately stage disease in these patients [12].

Radical cystectomy (RC) is the gold standard treatment for SCC of the bladder. Other management approaches, including pre-operative radiotherapy, followed RC, show to confer a survival advantage over RC alone, the latter demonstrating less frequency of local failures. Due to the rarity of this type of cancer and the small population studies, evidence for this approach is limited [13-15].

In a clinicopathologic analysis of 64 patients, SCC's survival rate was better with radical surgery than with chemotherapy or radiation; however, the outcome improved in tumors with advanced stage neoadjuvant radiation [16]. A study by Nishiyama et al., which included 1,131 patients with urothelial carcinomas and non-urothelial bladder who underwent RC, showed no difference in five-year survival [17]. Overall, not many studies can support the data available to define a prognosis of this condition due to a small number of patients in the studies and bias on patients' selections [16].

In this case, the patient's long history of CA with dysplasia was a significant risk factor. Interestingly his initial presentation with DVT was misleading, making the diagnosis challenging. Once the masses were identified and the aspirate illustrated atypical cells, these findings urged additional testing. The diagnosis of SCC, although incidental, was a fortunate finding and prevented a longer undiagnosed course.

## Conclusions

The progression of CA to SCC is rare, with an undetermined prognosis. The diagnosis tends to be challenging as it can present asymptomatically or with unspecific symptoms; hematuria is one of the most common, and other symptoms can be categorized as irritative or obstructive symptoms (nocturia, pelvic pain, dysuria, etc.) are less frequent, and usually indicates an advanced stage. We recommend that clinicians regularly follow up with patients with a CA history with a multidisciplinary approach and consider the potential progression to malignancy, as an incidental diagnosis can become prevailing.
